# Effects of Pilates on inter-recti distance, thickness of rectus abdominis, waist circumference and abdominal muscle endurance in primiparous women

**DOI:** 10.1186/s12905-023-02775-5

**Published:** 2023-11-27

**Authors:** Namee Lee, Young-Hyeon Bae, Shirley S. M. Fong, Wan-Hee Lee

**Affiliations:** 1 Department of Physical Therapy, Graduate School, Sahmyook University, Seoul, Republic of Korea; 2 Department of Healthcare and Public Health, National Rehabilitation Center, Seoul, South Korea; 3 Department of Health and Physical Education, Education University of Hong Kong, Hong Kong, Hong Kong, Special Administrative Region of China; 4 Department of Physical Therapy, Sahmyook University College of Health Science, Seoul, Republic of Korea

**Keywords:** Exercise, Primiparous, Rectus abdominis, Ultrasound, Diastasis rectus abdominis

## Abstract

**Background:**

Pilates is expected to have a positive effect on women with weakened abdominal muscles after childbirth. Pilates may have a beneficial effect on the structure and function of the abdominal muscles in pregnant women. Therefore, the objective of this study was to investigate the effects of Pilates on inter-recti distance, thickness of the rectus abdominis, waist circumference, and abdominal muscle endurance in primiparous women.

**Methods:**

Thirty-five primiparous postpartum women were assigned to either the Pilates exercise group (*n* = 20) or the control group (*n* = 15). Pilates was undertaken by the exercise group for 50 min/day, 5 days/week, for 4 weeks. The control group maintained their daily activities without any intervention. The inter-recti distance was measured at three locations along the linea alba, and the thickness of the rectus abdominis was measured using ultrasound. Abdominal muscle endurance was measured using a repeated 1-min curl-up test. Waist circumference was also measured.

**Results:**

The exercise group showed significant improvements from baseline in inter-recti distance, waist circumference, and abdominal muscle endurance (*p* < 0.05). The control group showed no significant improvement in these variables. Compared with the control group, the exercise group showed significantly improved performance in terms of inter-recti distance, waist circumference, and abdominal muscle endurance (*p* < 0.05).

**Conclusions:**

The results of this study demonstrate that Pilates was effective in reducing inter-recti distance and waist circumference and improving abdominal muscle endurance in primiparous postpartum women. Pilates is considered an effective exercise for improving muscle structure and function in primiparous postpartum women, helping in the recovery from, and preventing, diastasis rectus abdominis.

## Background

Physical changes resulting from pregnancy include the development of the mammary glandular tissue, breast enlargement, and enlargement of the uterus to 5–6 times its size before pregnancy, pushing the diaphragm upward in the abdominal cavity, and ultimately reducing the size of the thoracic cavity [1]. In addition, the mother’s center of gravity changes, lordosis of the lumbar region increases, and the binding between the ligament of the sacroiliac articulation and the pubic bone is softened and stretched under the influence of the hormone relaxin, which makes the joints more flexible and improves movement [2, 3]. These effects increase the pelvic cavity size and make delivery easier [1–3]. Owing to the elasticity changes in the connective tissue during pregnancy and the fetus displacing abdominal organs, the mechanical stress acting on the abdominal walls expands the linea alba, and the rectus abdominis muscle begins to separate from the 14^th^ week of pregnancy, increasing the most in the third trimester, and continuing until childbirth [3–5]. Moreover, an increase in the inter-recti distance during pregnancy lowers the stability of the pelvis by weakening the abdominal muscles and reducing their control [6–15]. This decrease in stability leads to dysfunction, pain, and incorrect posture [6–15].

Diastasis rectus abdominis occurs when the inter-recti distance becomes more than 2 cm at one or more of the navel levels or an upper or lower point of the navel. It has been reported that it naturally recovers within 8 weeks after childbirth, whereas it has been reported that in some cases, recovery was only 60.7% by 6 months after childbirth and for others had not recovered even after 1 year [6, 15–21]. A review on the management of diastasis rectus abdominis cases suggested that two-dimensional (2D) ultrasound imaging should be a reliable method for accurately diagnosing diastasis rectus abdominis in women during the postpartum period [6, 15–18, 20, 21]. In addition, intuitive ultrasound imaging feedback on the inter-recti distance while implementing exercise programs has been shown to help enhance correct movement in postpartum women [6, 22]. Recently, it was reported that ultrasound imaging was effective in evaluating the effect of reducing the inter-recti distance after a yoga exercise program [6].

Postnatal exercise is effective for the recovery of elasticity in the abdominal walls, pelvis, and muscles that increase during delivery, as well as for weight loss and alleviation of anxiety and depression [23–25]. This has a positive effect on physical fitness [24, 25]. Although diastasis rectus abdominis is a common and important clinical problem in women after childbirth, little is known about its prevention or management [26]. However, regular exercise before pregnancy prevents and alleviates diastasis rectus abdominis during pregnancy [26]. In addition, abdominal muscle strengthening exercise programs are prescribed for diastasis rectus abdominis; however, the effect of nonsurgical treatment, including exercise programs, on the prevention and reduction of diastasis rectus abdominis is unclear. Nevertheless, a core-based abdominal muscle-strengthening exercise program including a drawing-in movement that activates the transverse abdominis and internal abdominal oblique, an abdominal crunch that lifts the upper body by contracting the rectus abdominis muscle, and curl-up with transverse abdominis movement was shown to relieve linea alba distortion and reduce the inter-recti distance [4, 7–9, 12–15, 18, 27–29]. Therefore, it has been demonstrated that abdominal muscle strengthening exercise programs after giving birth, based on breathing and core training, improved the reduction of inter-recti distance when evaluated using ultrasound imaging [6].

Recently, there has been increasing interest in Pilates because of its effect on reshaping the body and improving physical fitness. Pilates, similar to yoga, are exercise programs that connect the deep muscles of the anterior abdominal wall and pelvic floor through breathing, and provide stability to the uterus with coordinated movement of the limbs [24, 30–34]. In addition, it was confirmed that the muscles of respiration and the anterior abdominal wall were activated after eight weeks of Pilates application in post-partum women [32]. Therefore, Pilates is expected to positively affect women with weakened abdominal muscles after childbirth. However, there is a lack of research on the application of Pilates in postpartum women, and no studies have revealed its effects on diastasis rectus abdominis. Therefore, it is important to study the effects of Pilates on the structure and function of abdominal muscles in postpartum women by investigating the reduction of the inter-recti distance and various other factors using ultrasound imaging. This study aimed to provide basic data from primiparous women who recovered from or prevented diastasis of the rectus abdominis through performing Pilates, by investigating the effects on inter-recti distance, rectus abdominis muscle thickness, waist circumference, and abdominal muscle endurance.

## Methods

### Participants

**Fig. 1:**
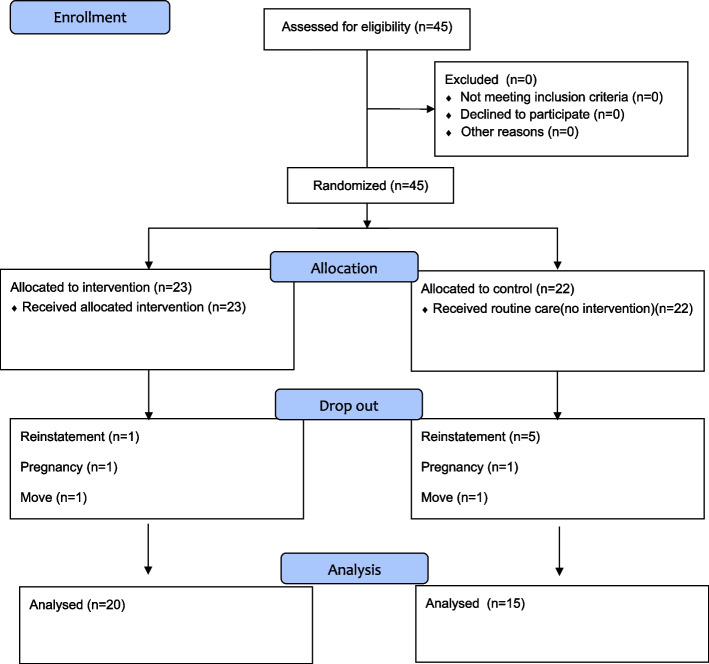
Experimental procedure

This study used snowball sampling because of difficulties in recruiting participants. Using this method, we recruited 45 primiparous women living in Seoul and Gyeonggi-do, South Korea. The number of participants required was calculated using G*Power 3.1. There was an expected group difference of 1%, a power of 0.8, and an alpha level of 0.05, indicating that 17 subjects were required per group. Allowing for a conservative dropout rate of 20%, we recruited 23 and 22 participants for the exercise and control groups, respectively. The inclusion criteria of the study subjects were women who were between 2 and 12 months postpartum and had vaginal delivery, or cesarean delivery with a Pfannenstiel incision at gestation between 37 and 42 weeks. Through a pre-survey, this study excluded those whose newborns weighed 2.5 kg or less at delivery, had more than one birth, had a multiple birth, had spinal deformities, underwent spinal surgery for low back pain, had diabetes or hypertension, had neurological or respiratory disorders, and had medical complications. All subjects were informed in advance about the requirements and objectives of the study and their right to withdraw from the study at any time, even after the study had begun, owing to personal circumstances. Subsequently, participants voluntarily submitted informed consent forms. During the exercise program, three people from the exercise group and seven people from the control group were eliminated, and 20 people from the exercise group and 15 people from the control group completed the exercise program and were included as the final analysis subjects (Fig. 1).

### Study procedure

This study used a pretest–posttest control group design with two groups. Subjects who met the inclusion criteria submitted a written consent form before the study, had a pre-examination three days before the start of the study, had four weeks of exercise, and had a post-examination the day after the end of the study. For pre- and post-evaluation, the inter-recti distance and rectus abdominis muscle thickness were measured using portable ultrasound to check for structural changes in the abdominal muscles. In addition, the waist circumference was measured with a tape measure, and the abdominal muscle endurance was measured through a dynamic abdominal muscle endurance test.

### Exercise intervention

In this study, the control group maintained daily life activities without intervention, whereas the exercise group performed the Pilates exercise program. Two Pilates instructors and former physiotherapists guided the exercises.

In the exercise group, the Pilates exercise program was performed for 50 min per session, five sessions a week for a total of four weeks, in which they performed group exercises three times a week and a self-exercise program twice a week. The self-exercise program consisted of movements learned during group exercise. After detailing the sequences, repetitions, and sets, the researcher sent a message containing a list of movements, photos, and videos to the participants on the day of the self-exercise. After each self-exercise session, the researcher asked the participants to report via messaging whether the exercises were fully completed, and encouraged participants to complete at least seven out of eight self-exercise sessions.

This study designed a Pilates exercise program to stabilize the waist and pelvic area and improve muscular endurance through use each week after childbirth, which ultimately aimed to restore the thoracic, abdominal, and pelvic muscles that became loose during pregnancy and the areas affected by surgery during cesarean section and vaginal delivery. The program was structured around the Pilates mat exercise program, which has been reported to decrease the inter-recti distance and be effective in strengthening the rectus abdominis muscle, considering the tension of the linea alba (Table 1).

**Table 1:** Pilates program for Primiparous women

Week	Week 1	Week 2	Week 3	Week 4
Intensity	RPE 10 ~ 12	RPE 10 ~ 12	RPE 12 ~ 14	RPE 14 ~ 16
Supine position	breathing pelvic clock toe taps pushing knee bridge curl up	breathing pelvic clock toe taps pushing knee bridge curl up diagonal curl up hundred one leg stretch	breathing pelvic clock toe taps bridge curl up diagonal curl up hundred one leg stretch single leg bridge	Breathing pelvic clock toe taps bridge diagonal curl up hundred one leg stretch crisscross single leg bridge
Prone position	breathing	breathing	breathing	breathing
Side-lying position	inside leg lift	inside leg lift double leg lift	double leg lift side push up	side push up
Quadruped position	breathing pelvic clock	breathing pelvic clock	breathing double knee off alternate arm & leg lift	breathing alternate arm & leg lift
Sitting position	breathing mermaid	breathing mermaid	breathing side bend 1	breathing side bend 2

*RPE* Rating of perceived exertion

The Pilates exercise program consisted of 10 min of warm-up, 30 min of the main exercise, and 10 min of warm-down. The positions during the exercise were taken in the order of the supine, prone, lateral recumbent, quadruped, and sitting positions. All exercises were performed with the trunk and pelvis stabilized using alert torso breathing and deep stabilizer muscles. The movements were modified and re-applied if the participant experienced pain or physical discomfort [24, 30–34].

**Fig. 2:**
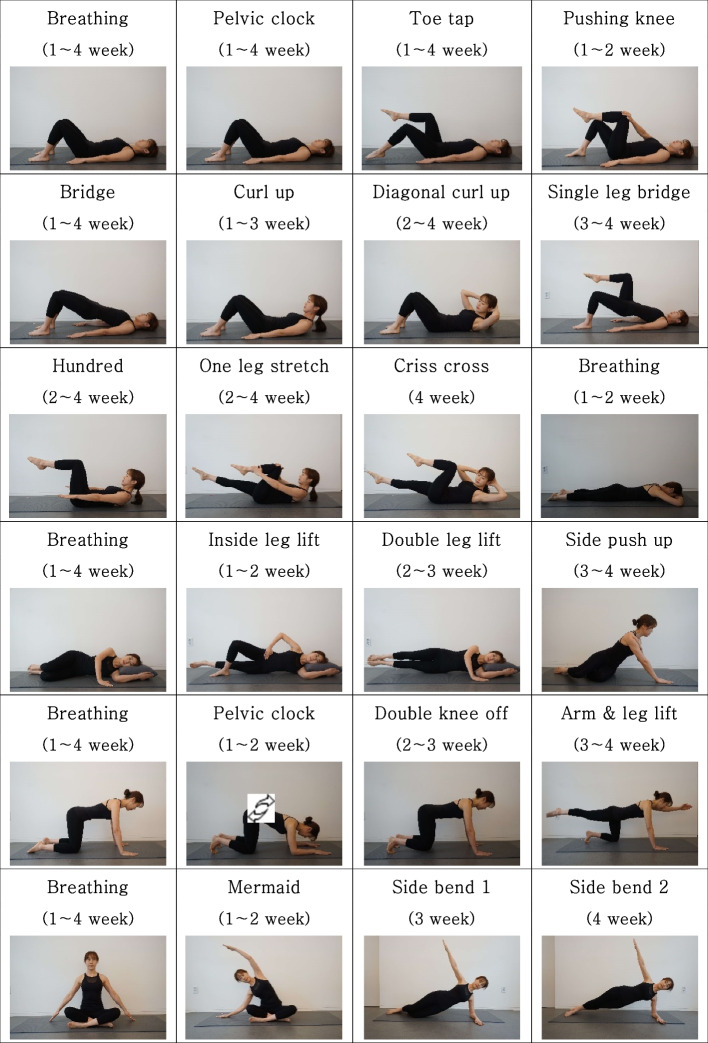
Pilates exercise program

Based on performing breathing exercises for respiratory muscles and abdominals to stabilize the pelvis in each posture, the Pilates exercise program included the hundred single-leg stretch for abdominals, back and hip extensors after isometric contraction of the abdominal muscles, and included curl-up, diagonal curl-up, and criss-cross after contraction of the transverse abdominis. In addition, the Pilates program used lumbar stabilization exercises such as a pelvic clock, toe tap, pushing knee, bridge, single leg bridge in supine position, double knee off, alternate arm and leg lift in quadruped position, inside leg lift, double leg lift, side push up, mermaid, and side bend to strengthen the abdominal oblique muscle (Fig. 2).

Exercise intensity was controlled using Borg's rating of perceived exertion (RPE). To set up the initial exercise intensity, the subjects were informed of the RPE concept and requested to provide feedback by expressing their subjective intensity on a scale of 1–20. The program was then adjusted on an average basis with a moderately weak-intensity of RPE 10–12. Every Friday, the subjective RPE was checked for each participant, and this was reflected in the composition of the exercise program for the following week. After an adaptation period of one week before starting, the Pilates exercise program was started with an RPE of 10–12 points (moderately weak) in weeks 1–2 and gradually increased to an RPE of 12–14 points (somewhat strong) in week 3 and RPE 14–16 points (strong) in week 4 by increasing the difficulty of movements according to the principle of progressive overload. One set was conducted, with each set consisting of repeating the motion maintained for 5–10 s, up to 10 times. A break of 45–60 s was provided between each exercise.

### Outcome measures

#### Assessment of inter-recti distance and thickness

**Fig. 3:**
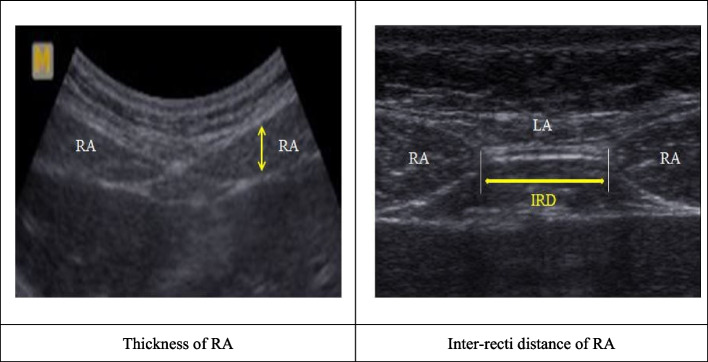
Measurement of inter-recti distance and thickness of rectus abdominis by ultrasound. RA: Rectus abdominis, LA: Linea alba, IRD: Inter-recti distance

A review on the management of diastasis rectus abdominis cases suggested that 2D ultrasound imaging should be a reliable method for accurately diagnosing diastasis rectus abdominis in women during the postpartum period [6, 15–18, 20, 21]. To assess the inter-recti distance, various methods can be used, such as measuring two points, including the navel and the midpoint between the navel and xiphisternum; measuring three points, including the point at 2 or 5 cm above the navel, the point 2 or 5 cm below the navel, and the navel; and measuring four points, including the points at 5 cm above the navel, 2 cm above the navel, and 2 cm below the navel, and the navel [7, 17, 20, 28]. Since diastasis rectus abdominis appears differently depending on the location of each rectus abdominis muscle, and most symptoms occur at the navel level, it is important to measure the navel and its surroundings by location. In addition, the longitudinal width of the linear probe of the portable ultrasound 2D B-mode used in this study was 1.5 cm [3]. Considering these, this study measured three points, at 4.5 cm above the navel, at 4.5 cm below the navel, and directly below the navel. In the supine position, the participants placed the soles of their feet on the floor with their legs bent, a pillow under their head, and both arms extended next to the trunk. For the measurements, the linear probe was located transversely along the linea alba in the midline of the trunk, and measurements were made at the three points in the abdominal muscle relaxation position while exhaling. In addition, the inter-recti distance was measured at the median point connecting the boundaries of the fascia of the rectus abdominis muscle on both sides [16, 20] (Fig. 3). All measurements were performed by a specially trained examiner, and the mean value of three repeated measurements was used as the measurement value in conjunction with Digital Imaging and Communication in Medicine (DICOM). The reliability within the examiner measuring the inter-recti distance by ultrasound showed an intra-class correlation coefficient (ICC) of 0.72–0.91 during abdominal relaxation [20, 21, 29]. To evaluate structural changes in the abdominal muscle, the thickness of the rectus abdominis muscle was measured using a curved probe in portable ultrasound 2D B-mode. The measurement positions were the same as those used to measure the inter-recti distance. The curve-type probe was located transversely along the linea alba in the midline of the trunk, and measurements were made at the navel’s lower point in the abdominal muscle relaxation position while exhaling. The thickness of the rectus abdominis muscle was measured as the line connecting perpendicularly between the upper boundary point, which is shown as a white image at an inner point of 2.0 cm concerning the right rectus abdominis muscle and the lower boundary point of the fascia. One examiner performed all measurements, and the mean value of three measured images was used as the measurement value in conjunction with DICOM [20, 21, 29].

### Assessment of waist circumference and abdominal muscle endurance

Waist circumference was measured at the navel, using a tape measure with the subject in an upright position [35]. The subject stood upright with both hands crossed over the chest, while the examiner stood in front of the subject, and accurately positioned the tape measure using the cross technique. The subject breathed naturally with her arms down, and measurements were taken while relaxing and exhaling. The measurement units were in centimeters and recorded to the first decimal place.

To measure abdominal muscle endurance, the subjects supported their feet on the floor while bending their knee and hip joints in the supine position. The head rested on a pillow that fitted the height of the head, and the palms were placed on the ground with the arms extended. After lifting the head and shoulders 30–40° from the floor, the subject lifted and held their hands for an additional 8 cm and then returned to the original position so that the shoulders touched the floor. At this point, the chin remained horizontal, not pointing up. After setting the metronome to hold at 40 beats per minute, the subject started at the first beat and returned to the next beat. The number of times the subject performed the task was counted, in which the cycle of raising the torso and returning to the original position was considered once. If the participant’s shoulders did not touch the ground when returning or if they could not match the speed of the metronome beats, they were instructed to stop the movement. The reliability of within-examiner measurement of abdominal muscle endurance showed an ICC of 0.89 [36].

### Data analysis

The Shapiro–Wilk test was used to confirm the general characteristics of the subjects and the normality test for the variables, while the chi-square test and independent samples t-test were used for the intergroup homogeneity test. Changes in the dependent variables before and after the Pilates exercise program were analyzed using a paired t-test. An independent samples t-test was used to compare the effects between the groups. The statistical significance (α) of all data was set at 0.05.

## Results

Comparative analysis of the general and physical characteristics of the 20 subjects in the Pilates exercise group and the 15 subjects in the control group at the baseline showed that the two groups had no significant differences (*p* > 0.05) in age, height, weight, body mass index, neonatal weight, months of pregnancy at childbirth, delivery method, inter-recti distance, rectus abdominis muscle thickness, waist circumference, or abdominal muscle endurance, indicating that the two groups were homogeneous (Tables 2 and 3).

**Table 2:** General characteristics of subjects

Variable	Exercise group (mean ± SD)	Control group (mean ± SD)	t / X^2^	*p*
Age (year)	34.10 ± 2.94	33.87 ± 3.91	0.202	0.841
Height (cm)	163.40 ± 4.00	162.00 ± 5.95	0.832	0.411
Weight (kg)	56.54 ± 4.69	56.80 ± 6.86	-0.133	0.895
BMI (kg/m^2^)	21.17 ± 1.45	21.68 ± 2.73	-0.720	0.496
Neonatal weight (kg)	3.24 ± 0.37	3.26 ± 0.32	0.201	0.842
Postpartum period (Month)	6.45 ± 2.23	5.73 ± 2.69	0.861	0.395
Natural childbirth / caesarean section (n)	10 / 10	10 / 5	0.971	0.339

*BMI* Body mass index, *SD* Standard deviation

**Table 3:** Physical characteristics of subjects

Variable		Exercise group (mean ± SD)	Control group (mean ± SD)	t	*p*
IRD	Above 4.5 cm the navel	1.27 ± 0.41	1.06 ± 0.42	1.498	0.144
	Navel	1.77 ± 0.52	1.48 ± 0.52	1.623	0.114
	below 4.5 cm the navel	1.19 ± 0.53	0.94 ± 0.42	1.504	0.142
Thickness of RA		1.03 ± 0.88	0.98 ± 0.09	1.728	0.093
Waist Circumference		83.18 ± 5.56	85.68 ± 7.18	-1.165	0.252
Abdominal Endurance		11.00 ± 6.74	8.00 ± 5.98	1.367	0.181

*IRD* Inter-recti Distance

In the exercise group, there were significant changes after the intervention in inter-recti distance, waist circumference, and abdominal muscle endurance (*p* < 0.05), but no difference was observed in the rectus abdominis muscle thickness. In the control group, no significant changes were observed in any of the variables during the study period (Table 4). The exercise group showed a significant reduction in the inter-recti distance compared to the control group (*p* < 0.05). Regarding waist circumference, the exercise group showed a significant reduction compared to the control group (*p* < 0.05). In addition, the exercise group showed a significant improvement in abdominal muscle endurance compared with the control group (*p* < 0.05). However, rectus abdominis muscle thickness was not significantly different between the two groups (Table 5).

**Table 4:** Comparison between before and after intervention in each groups

Variable			Before (mean ± SD)	After (mean ± SD)	t(*p*)
IRD	Above 4.5 cm the navel	Exercise group	1.27 ± 0.41	0.97 ± 0.51	-3.725(0.001)^*^
		Control group	1.06 ± 0.42	1.00 ± 0.42	-1.180(0.258)
	Navel	Exercise group	1.77 ± 0.52	1.16 ± 0.56	-4.913(0.001)^*^
		Control group	1.48 ± 0.52	1.30 ± 0.39	-1.905(0.077)
	below 4.5 cm the navel	Exercise group	1.19 ± 0.53	0.53 ± 0.15	-6.503(0.001)^*^
		Control group	0.94 ± 0.42	0.84 ± 0.40	-1.996(0.066)
Thickness of RA		Exercise group	1.03 ± 0.88	1.06 ± 0.15	1.187(0.250)
		Control group	0.98 ± 0.09	1.00 ± 0.09	1.318(0.209)
Waist Circumference		Exercise group	83.18 ± 5.56	80.33 ± 5.89	-4.156(0.001)^*^
		Control group	85.68 ± 7.18	85.13 ± 6.91	-0.861(0.404)
Abdominal Endurance		Exercise group	11.00 ± 6.74	19.55 ± 1.23	5.474(0.001)^*^
		Control group	8.00 ± 5.98	9.27 ± 8.31	0.921(0.372)

*IRD* Inter-recti Distance, *RA* Rectus abdominis, *SD* Standard deviation

^*^p < 0.05

**Table 5:** Comparison of mean difference between groups

Variable	Exercise group (mean ± SD)	Control group (mean ± SD)	t(*p*)
IRD 4.5 cm above the navel (cm)	-0.30 ± 0.36	-0.06 ± 0.19	-2.346(0.025)^*^
IRD below the navel (cm)	-0.61 ± 0.55	-0.18 ± 0.37	-2761(0.011)^*^
IRD 4.5 cm below the navel (cm)	-0.65 ± 0.45	-0.10 ± 0.19	-4.474(0.001)^*^
Thickness of RA (cm)	0.03 ± 0.11	0.02 ± 0.05	0.412(0.683)
Waist Circumference (cm)	-2.85 ± 3.07	-0.55 ± 2.49	-2.445(0.020)^*^
Abdominal Endurance(number)	8.55 ± 6.99	1.27 ± 5.32	3.500(0.001)^*^

*IRD* Inter-recti Distance, *RA* Rectus abdominis, *SD* Standard deviation

^*^p < 0.05

## Discussion

Recently, many studies have verified the effectiveness of various exercise programs for relieving symptoms of the rectus abdominis muscle after childbirth [4, 6, 7, 9, 12–15, 29, 37, 38]. In previous studies, diastasis rectus abdominis was gradually relieved over time, and the inter-recti distance naturally recovered within eight weeks in most cases [3, 16]. Therefore, this study recruited subjects 2–12 months after childbirth to reduce the influence of factors that might affect natural postnatal recovery, and to ensure the homogeneity of general and physical characteristics between the intervention and control groups. The aim of the study was to test the effectiveness of a Pilates exercise program for the recovery and health care of women after childbirth. It has been shown that the quantitative evaluation of the inter-recti distance by ultrasound imaging is reliable, valid, and a simple method to diagnose diastasis rectus abdominis early after childbirth [17, 19]. Furthermore, intuitive feedback on changes in inter-recti distance using ultrasonic image evaluation may strengthen the instructions of the exercise program [17, 19]. Therefore, this study measured the inter-recti distance and rectus abdominis muscle thickness using ultrasonic imaging to evaluate the changes of the rectus abdominis, and measured waist circumference and abdominal muscle endurance to check health management effects before and after the application of the Pilates exercise program [21].

Targeting primiparous women 2–12 months postpartum, this study included an exercise group of 20 participants undertaking a Pilates exercise program and a control group of 15 participants who maintained daily life without intervention. A comparative analysis of the changes after the intervention period showed a reduction in the inter-recti distance, waist circumference, and abdominal muscle endurance in the exercise group compared to the control group. However, there was no significant difference in muscle thickness between the exercise and control groups.

In a previous study that applied an abdominal and pelvic floor muscle exercise program to women after childbirth, the inter-recti distance was significantly different between the exercise and control groups [15], supporting the results of this study. It is postulated that these positive results from our study can be attributed to the application of the Pilates exercise program, involving 20 sessions over 4 weeks, leading to abdominal muscle contraction, which caused a reduction in the abdominal horizontal diameter and subsequently generated a horizontal force that reduced the inter-recti distance. In a previous study that applied a breathing and core-based abdominal muscle strengthening exercise program for a total of 24 sessions with a frequency of three sessions per week for eight weeks, the overall inter-recti distance was significantly reduced [6]. In contrast, a study that applied a total of 12 sessions with a frequency of one session per week for 12 weeks as an exercise program found no significant reduction in the inter-recti distance below the navel compared to the control group [6]. Considering these results, it is believed that the weekly frequency of the applied exercise program and the total number of sessions should affect the reduction in inter-recti distance [15, 35].

In a study that analyzed the thickness and changes in the rectus abdominis, transverse abdominis, external oblique, internal oblique, multifidus, and erector spinae muscles for six weeks, significant improvements were observed in all muscles [32]. In our study, muscle thickness increased after four weeks in both the exercise and control groups, although the increase was not statistically significant, and there was no significant difference between the groups. Previous studies have suggested a minimum period of six weeks to change the thickness in the abdominal muscle. Our study intervention was only for four weeks, which may have been too short a period to elicit a significant change through the Pilates exercise program.

During pregnancy, the level of free fatty acids in the blood increases 2–4 times, and approximately 3.5 kg of fat accumulates [39]. Waist circumference is considered a measure of abdominal obesity, and a pregnant woman's weight increases during pregnancy [23, 39]. Aerobic exercise is a direct method of burning body fat, and anaerobic exercise is a method of increasing basic metabolism to reduce body weight and fat [23, 39]. Nonetheless, regular and continuous exercise, regardless of the type, reduces body fat in both obese and normal individuals [23, 39]. Other studies have shown that after a 12 week Pilates exercise program by mothers who underwent natural births, the exercise group showed a significantly greater decrease in waist circumference than the control group without exercise [10, 24, 30, 31, 33, 34]. In this study, the exercise group showed a significant decrease after four weeks. In the control group, waist circumference decreased, but the difference was not statistically significant. Natural recovery was believed to have occurred in the control group.

Childbearing women naturally experience a decrease in muscular endurance owing to anatomical and physiological changes, including hormonal changes. When a previous study compared postpartum women at the 6^th^ month after childbirth with non-postpartum women, there was a significant difference in the muscular strength of trunk rotation, static endurance of trunk flexion and rotation, and dynamic endurance of trunk flexion and rotation. Moreover, there was a correlation between reduced inter-recti distance and improved function of abdominal muscles [18]. In this study, a significant improvement in muscular endurance was observed in the exercise group. The control group also showed improvement, but the difference was not statistically significant. There was a significant difference between the two groups. After the Pilates exercise program, the inter-recti distance decreased and muscular endurance improved.

This study investigated the effects of a Pilates exercise program based on breathing and core training on inter-recti distance, rectus abdominis muscle thickness, waist circumference, and abdominal muscle endurance in women who had undergone their first childbirth. The study showed that the Pilates exercise program significantly improved the inter-recti distance, waist circumference, and abdominal muscle endurance in primiparous women, and the Pilates exercise program is recommended as a way to recover from or prevent diastasis of the rectus abdominis in postpartum women.

However, this study had some limitations. Since the study was conducted in women at an average of five months after childbirth, it is still necessary to provide a quality randomized controlled trial that demonstrates that the Pilates exercise program can reduce the inter-recti distance early in the puerperium, taking into account the individual's natural recovery. In addition, this study was limited to showing the relationship between the change in inter-recti distance and physical function changes, such as rectus abdominis muscle thickness, waist circumference, and abdominal muscle endurance [6, 11, 27, 29]. In future studies, it will be necessary to measure the degree of deformation of the linea alba and the inter-recti distance in parallel to increase reliability when evaluating the symptoms of separation of the rectus abdominis muscles. To apply the systematic Pilates exercise program to women after childbirth, the effect of the long-term postnatal exercise program should be verified, and the influence of daily lifestyle or posture on the exercise program should be further studied.

## Conclusion

The results of this study are consistent with those of a previous study. Based on the improvement in inter-recti distance, waist circumference, and abdominal muscle endurance, the Pilates exercise program, which strengthens the abdominal muscles based on breathing and core training, is recommended as an effective exercise for improving muscle structure and function in primiparous women to recover from or prevent diastasis rectus abdominis.

## Data Availability

The datasets used and/or analyzed during the current study are available from the corresponding author on reasonable request.
